# Public Health Responses to Reemergence of Animal Rabies, Taiwan, July 16–December 28, 2013

**DOI:** 10.1371/journal.pone.0132160

**Published:** 2015-07-10

**Authors:** Angela Song-En Huang, Wan-Chin Chen, Wan-Ting Huang, Shih-Tse Huang, Yi-Chun Lo, Sung-Hsi Wei, Hung-Wei Kuo, Pei-Chun Chan, Min-Nan Hung, Yu-Lun Liu, Jung-Jung Mu, Jyh-Yuan Yang, Ding-Ping Liu, Jih-Haw Chou, Jen-Hsiang Chuang, Feng-Yee Chang

**Affiliations:** 1 Office of Preventive Medicine, Centers for Disease Control, Taipei, Taiwan; 2 Epidemic Intelligence Center, Centers for Disease Control, Taipei, Taiwan; 3 Center for Research and Diagnostics, Centers for Disease Control, Taipei, Taiwan; 4 Office of Deputy Director, Centers for Disease Control, Taipei, Taiwan; 5 National Defense Medical Center, Tri-Service General Hospital, Taipei, Taiwan; Thomas Jefferson University, UNITED STATES

## Abstract

Taiwan had been free of indigenous human and animal rabies case since canine rabies was eliminated in 1961. In July 2013, rabies was confirmed among three wild ferret-badgers, prompting public health response to prevent human rabies cases. This descriptive study reports the immediate response to the reemergence of rabies in Taiwan. Response included enhanced surveillance for human rabies cases by testing stored cerebrospinal fluids (CSF) from patients with encephalitides of unknown cause by RT-PCR, prioritizing vaccine use for postexposure prophylaxis (PEP) during periods of vaccine shortage and subsequent expansion of PEP, surveillance of animal bites using information obtained from vaccine application, roll out of preexposure prophylaxis (PrEP) with vaccine stock restoration, surveillance for adverse events following immunization (AEFI), and ensuring surge capacity to respond to general public inquiries by phone and training for healthcare professionals. Enhanced surveillance for human rabies found no cases after testing 205 stored CSF specimens collected during January 2010–July 2013. During July 16 to December 28, 2013, we received 8,241 rabies PEP application; 6,634 (80.5%) were consistent with recommendations. Among the 6,501persons who received at least one dose of rabies vaccine postexposure, 4,953 (76.2%) persons who were bitten by dogs; only 59 (0.9%) persons were bitten by ferret-badgers. During the study period, 6,247 persons received preexposure prophylaxis. There were 23 reports of AEFI; but no anaphylaxis, Guillain-Barré syndrome, or acute disseminated encephalomyelitis were found. During the study period, there were 40,312 calls to the Taiwan Centers for Disease Control hotline, of which, 8,692 (22%) were related to rabies. Recent identification of rabies among ferret-badgers in a previously rabies-free country prompted rapid response. To date, no human rabies has been identified. Continued multifaceted surveillance and interministerial collaboration are crucial to achieve the goal of rabies-free status in Taiwan.

## Introduction

Taiwan had been free of indigenous human and animal rabies case since canine rabies was eliminated in 1961 [1, 2], despite the lower than recommended 70% rabies vaccine coverage among dogs and cats. The successful maintenance of rabies-free status was attributed to Taiwan’s relative geographic isolation as an island and strict animal importation control [2].

However, human rabies remains nationally notifiable in Taiwan. Since the last locally-acquired human rabies case was confirmed in 1959, three imported cases of human rabies have been identified, including a female traveler from China (July 2002), a businessman returning from China (July 2012), and a male worker from the Philippines (May 2013), all of which occurred after dog bites outside Taiwan without receiving rabies postexposure prophylaxis (PEP) [3–5].

The Taiwan Council of Agriculture (COA) conducts surveillance of animal rabies by histopathologic examination of animal brain tissues using direct fluorescent antibody (DFA) testing and immunohistochemistry (IHC) [6–9]. No rabies virus were found among 6,841 dog specimens tested during 1999–2012 and 322 bat specimens tested during 2008–2012 [10].

**Fig 1:**
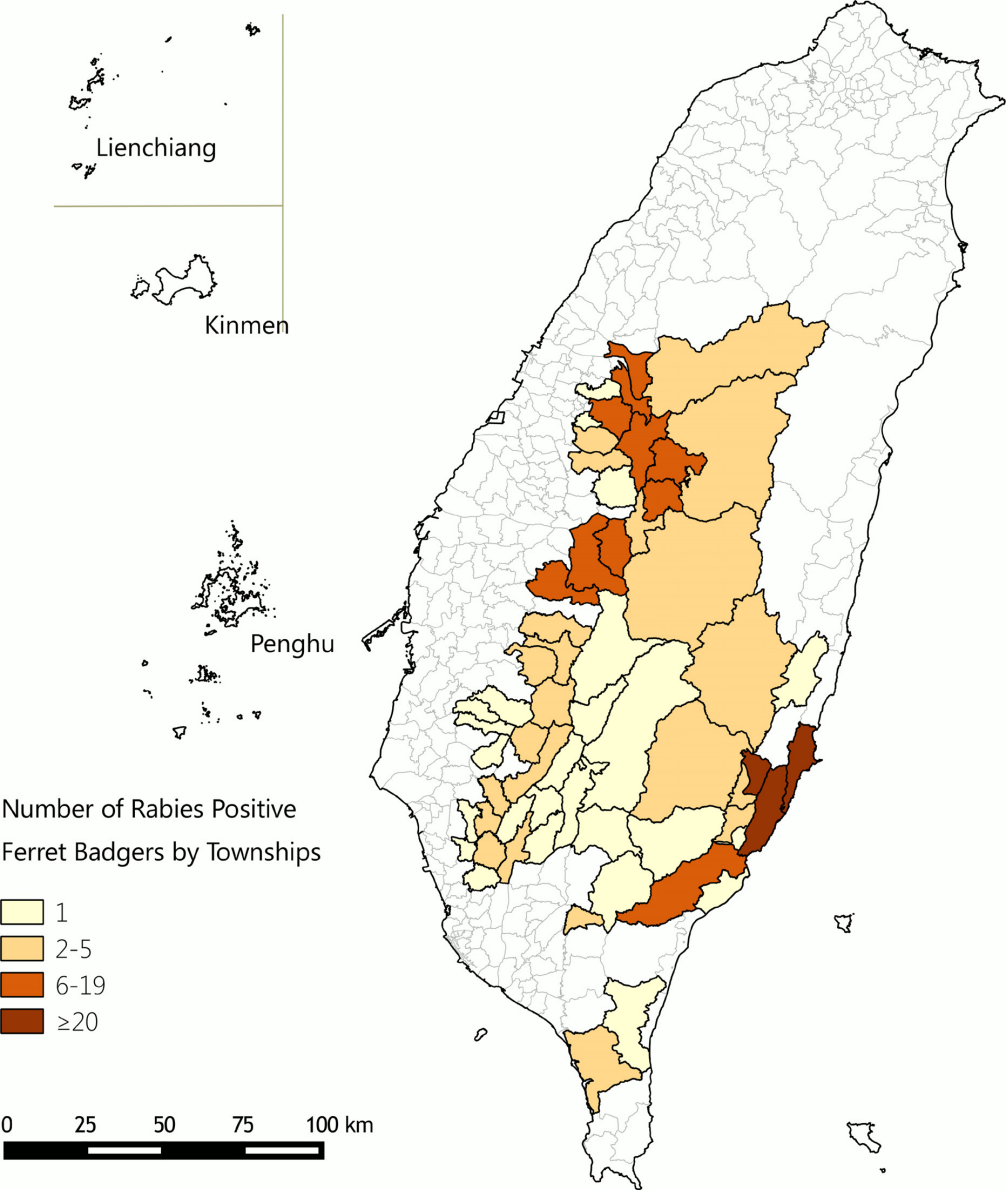
Distribution of rabies positive ferret-badgers, July 16–December 27, 2013 (n = 273). Rabies positive ferret-badgers found in colored areas.

In 2012, COA contracted two universities to conduct surveillance of emerging infectious diseases among wild animals; rabies real-time reverse transcription-polymerase chain reaction (RT-PCR) testing was added in 2013. Three dead ferret-badgers with unexplained encephalitides found in central Taiwan during May to December 2012 underwent a series of diagnostic tests, eventually tested positive for rabies virus by RT-PCR on June 24, 2013. The specimens were submitted to COA on June 26 for confirmatory testing. On July 16, COA confirmed rabies in these three ferret-badgers based on positive RT-PCR, DFA, and IHC results [1]. Of the 827 dead or behaviorally abnormal ferret-badgers collected during July 16 to December 27, 2013, rabies was confirmed by DFA in 273 animals found in central, southern and eastern Taiwan (Fig 1) [10]. In addition, COA confirmed rabies in a house shrew on July 30 and in a dog bitten by a rabid ferret-badger on September 10 [11]. Furthermore, three laboratory-confirmed rabies cases were found after testing 11 stored ferret-badger brain specimens collected during July 2010 to August 2012 [12].

In response to the reemergence of animal rabies, the Ministry of Health and Welfare and COA established an interministerial rabies working group on July 24 for integrated public health and agricultural response [13]. The Central Epidemic Command Center, chaired by the Vice Premier, was established on August 1 to coordinate interministerial efforts against rabies [14]. This report summarizes surveillance activities and immediate public health response to rabies reemergence in Taiwan.

## Methods

After the first rabid ferret-badger was identified, initial response focused on providing adequate supply of rabies vaccine and rabies immunoglobulin (RIG) for PEP [15]. Surveillance of rabid animals was established to respond to this reemerging rabies by COA [13]. Pathology and RT-PCR was performed to screen rabies virus among dead mammals and mammals with aggressive behavior, collected by forestry workers, wildlife rescue centers and local animal health agencies. Positive results were confirmed using DFA. Priorities for testing were given to animals with aggressive behavior and those which had bitten humans. Rabies test results were provided to Taiwan Centers for Disease Control (TCDC) daily so public health practitioners could follow-up on bite victims, ensuring the completeness of PEP [14]. Control measures implemented include expanded animal rabies surveillance, animal bite surveillance, revision of human rabies vaccine and RIG recommendations, expanded human rabies PEP service points, health education for the public and mammal bite care training for healthcare professionals.

### Enhanced surveillance of human rabies

Human rabies has been a notifiable disease in Taiwan since 1952. Rabies surveillance mandates physicians to notify clinically suspected cases to the health authority within 24 hours of diagnosis and submit clinical specimens including saliva, serum and cerebrospinal fluid (CSF) to TCDC laboratories. Rabies is confirmed if any of the specimens tested positive for rabies virus by diagnostic RT-PCR targeting both L and N genes of Rabies virus [16]. PCR products were sequenced in both directions to characterize the rabies amplicon. In addition, specific anti-rabies antibody testing by enzyme-linked immunosorbent assay [17] was performed in unvaccinated individuals. In addition, TCDC has conducted sentinel surveillance of human encephalitis of undetermined etiology since 2010. Physicians from 46 sentinel hospitals were requested to submit serum, throat swabs and CSF from patients with either encephalopathy or ataxia of unknown etiologies, plus any one of the following: fever, seizure, focal neurological signs, abnormal CSF profile, abnormal electroencephalography (EEG) and brain images. Submitted specimens were tested for viruses using a multiplex PCR panel containing probes targeting viruses causing encephalitis. Retrospective testing to detect rabies virus was conducted on stored CSF taken during January 1, 2010 to July 15, 2013, by rabies RT-PCR. The multiplex real-time PCR detected the following pathogens using primers designed based on previously published sequences: Herpes simplex virus 1 and 2 [18], VZV [18], CMV [18], Human Herpesvirus 6 and 7 [18], Hendra virus [19], Nipah virus [20], Dengue virus [21], chikungunya virus [22], Japanese encephalitis virus [23], West Nile virus [23], adenovirus [24], bocavirus [24], coronavirus (229E, HKU1, OC43 and NL63) [25], enterovirus [24], human metapneumovirus [26], human parechovirus [27], influenza virus (types A [28] and B[29]), parainfluenza virus (types 1–3, 4A and 4B) [24] [25], parvovirus B19 [30], polyomavirus (JC and BK) [31], respiratory syncytial virus (RSV) [29] and rhinovirus [24].

### Surveillance of mammal bites

Surveillance of mammal bites is conducted through a newly established PEP application system. Because of the initial shortage of RIG and rabies vaccines, distribution was tightly controlled by TCDC. Physicians needing to provide patients with RIG or rabies vaccines must send in their requests directly to TCDC by fax or email for review. Physicians must indicate biting animals, whether the animal was wild or owned, bite site on body of the bitten, bite category, according to WHO classification, circumstances of the incident, and the disposition of the animal that bit. Medical officers in TCDC would then review the application, and decide on whether to approve the application for PEP. Approval to provide treatment is given by phone within 30 minutes. After one month, as physicians have become familiar with rabies PEP recommendations, physicians were asked to provide rabies PEP immediately, and have their requests reviews retrospectively, to ensure that all patients needing rabies PEP were given appropriate PEP.

After the reemergence of animal rabies, the Advisory Committee on Immunization Practices (ACIP) in Taiwan established new recommendations of rabies PEP. During July 18–29, ACIP recommended PEP for individuals with category II or III exposure, as defined by the World Health Organization [32, 33], to wild mammals nationwide and stray dogs and cats in affected mountainous townships. PEP recommended include five doses of rabies vaccine given on days 0, 3, 7, 14, and 28, plus 20 IU/kg of human RIG given within 7 days of the first rabies vaccine dose at the wound site. On July 31, ACIP expanded PEP recommendations to individuals exposed to stray dogs and cats nationwide. RIG was initially prioritized for individuals with category III [33] exposure to ferret-badgers nationwide and to house shrews in Taitung. On September 6, with increased availability of RIG, individuals with category II exposure to ferret badgers in which rabies was confirmed by DFA were also recommended for RIG, to ensure that no human rabies occurred, even though this is contrary to WHO recommendation.

### Stockpile and distribution of vaccines

Government-funded PEP based on ACIP recommendations has been available through TCDC-contracted hospitals to individuals bitten by mammals. Before the reemergence of rabies in Taiwan, rabies preexposure prophylaxis (PrEP) and PEP were provided through 12 designated travel clinics to travelers. During 2010 to 2012, approximately 250 doses of rabies vaccines and 10 doses of RIG (HyperRAB S/D, [Talecris Biotherapeutics]) were administered annually. To provide easy access for all persons exposed, rabies PEP service points were expanded to 28 hospitals by the end of July 2013, and further expanded to 60 hospitals by early August. International procurement of vaccines was expedited with the support of Taiwan Food and Drug Administration (TFDA). Because of possible human RIG shortage, 2000 vials of 5 ml equine RIG (RabAvert [Novartis]) was also imported in August. PrEP programs began on July 27, targeting high risk groups, which included dog catchers, game wardens, animal shelter workers and veterinarians conducting rabies surveillance. Characteristics of all rabies immunization recipients, including demographics of the recipients, animal types and classification of animal-bite wound, were registered in a web-based system.

### Retrospective identification of ferret-badger exposures

From August 1 to September 30, 2013, persons exposed to ferret-badgers during July 2010 to July 2013 were urged to contact TCDC. Patients were asked about their age; sex; date, place, body part and extent of exposure and injury; history of vaccination against rabies; and phone number. Information was used for individual risk assessment of potential rabies exposure and issuance of PEP. Because no patients had fresh wounds, categorizing exposure based on WHO classification were based only on history.

### Monitoring adverse events following administration of rabies biologics

The national passive vaccine safety surveillance system, collaboratively operated by TCDC and TFDA, has served as a tool for adverse event following immunization (AEFIs) collection and vaccine safety signal detection since the 2009 influenza pandemic [34]. All reports of adverse events following RIG or rabies vaccines received during July 16–December 28, 2013 were reviewed and AEFIs coded with Medical Dictionary for Regulatory Activities (MedDRA) terms [35]. A report was categorized as serious when the patient outcome involved death, life-threatening illness, hospitalization, prolongation of an existing hospitalization, permanent disability, or congenital anomaly [36]; all other reports were considered nonserious. TCDC provided advice and assistance on the management of potentially serious adverse events (SAEs) for subsequent rabies PrEP or PEP vaccine doses.

### Health education, public health inquiries, and healthcare professional training

Multiple press releases and conferences were conducted educating the public on the dangers of animal bites and the management following bites. For additional information, anyone can call “1922”, a toll-free hotline set up by TCDC, using any landline or mobile phones. All inquiries were recorded automatically. We collected rabies-related inquiries from the record. The inquiries from July 16 to December 28, 2013 were included for analysis.

Healthcare professional also received training on animal bite wound management, evaluation of animal exposures for PEP using rabies vaccine and RIG, and evaluation of high risk groups for PrEP. Rapid development and roll out of training sessions were conducted by TCDC, providing information to healthcare workers both in hospitals and in the public health sector.

### Ethics

Specimen submitted by sentinel hospitals were collected previously for encephalitis surveillance and re-tested for rabies virus. Information collected from persons requiring rabies postexposure prophylaxis was for public health use. All specimen and information collection were done by the pronouncement of the Central Epidemic Command Center, and, in accordance with regulations of Taiwan, therefore did not require institutional review board approval. Only oral consent was obtained for specimens collected for surveillance purposes. The data was de-identified prior to analysis.

## Results

### Enhanced surveillance of human rabies

During July 16–December 28, 2013, there were no human rabies cases reported. During January 2010–July 2013, TCDC was notified of 365 cases with encephalitis of undetermined etiologies. Of the 205 available stored CSF specimens, none tested positive for rabies virus.

### Surveillance of mammal bites

**Fig 2:**
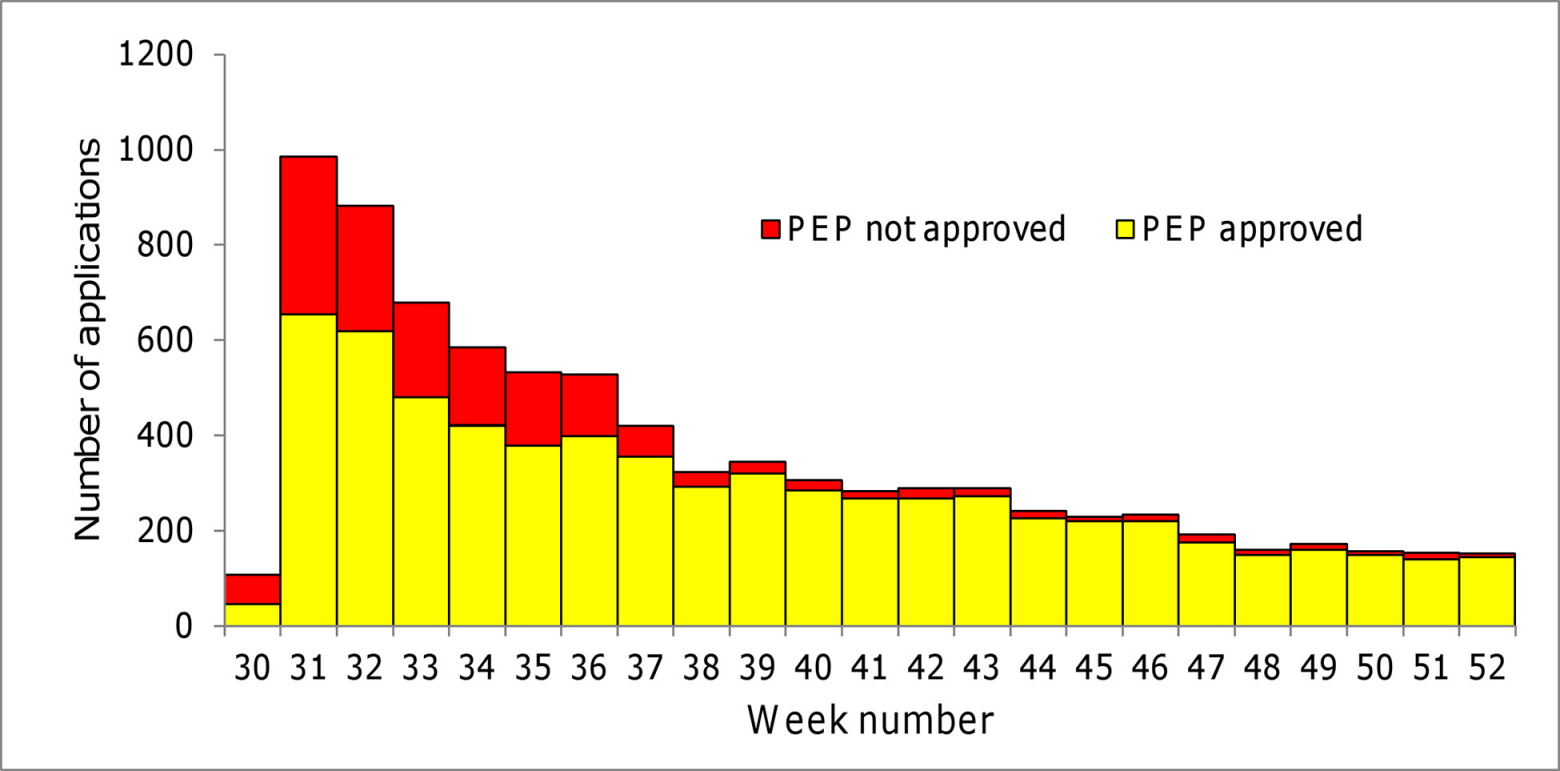
Rabies postexposure prophylaxis applications (n = 8,241) and public inquiries to Taiwan Centers for Disease Control hotline for information on rabies, July 14–December 28, 2013 (n = 8,692).

**Fig 3:**
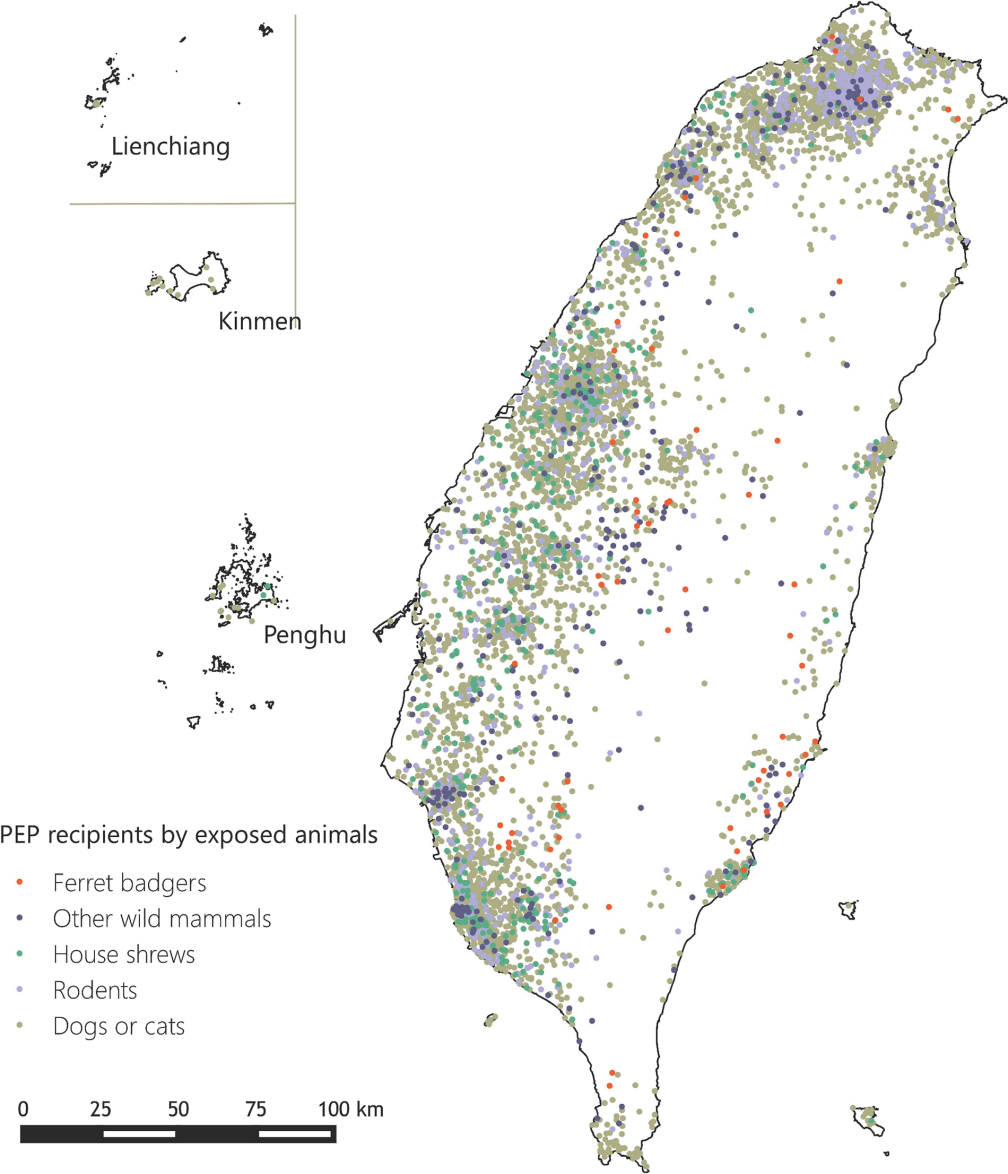
Geographic distribution of animal bites exposure location, July 16–December 28, 2013.

From July 17 to December 28, 2013, TCDC received requests for rabies PEP from 8,241 individuals. The peak of requests occurred on the week of July 28, two days after rabies was confirmed in a house shrew (Fig 2), and the median number of PEP requests per week was 289 (range, 107–985). PEP was denied in 1,607 (20%). The most common reasons for denial included category II or III wounds inflicted by pet dogs or cats (n = 1,245) and exposure as category I (n = 42). Persons sustained bites requiring PEP concentrated in coastal urban areas. Persons exposed to ferret-badger bites were distributed all over the island, and none in the offshore islands (Fig 3).

Of the 6,634 requests approved for PEP, 6,501 (98%) received at least one dose of vaccination. The median age of the PEP recipients was 42 years (range, 0–94), and 3,642 (56%) were male. The earliest date of animal exposure was February 16, 2011, as determined by retrospective identification of ferret-badger exposures, and the median interval from exposure to PEP application was 0 days (range, 0–896). Among those who received PEP, the most commonly exposed animals were dogs or cats (n = 4,953, 76%), followed by rodents (n = 893, 14%), house shrews (n = 406, 6%) and other wild mammals (n = 223, 3.4%). Category II exposures consisted of 2,855 applicants (43.9%) and category III in 3,613 applicants (55.6%). Ferret-badgers accounted for 59/223 (26%) of the wildlife exposures; among them, 37 (63%) persons had category III exposure, and 32 received RIG. Eight persons, even though they had category III exposure and RIG requests consistent with ACIP recommendations, did not receive RIG following physician evaluation. Physicians who chose to not give RIG were not asked for their reasons for doing so.

By February 24, 2014, there were 6501 persons who received at least one dose of PEP vaccination. Among them, 5,692 persons received at least three doses; 4,861 persons completed the first three doses within 7 days of starting the first dose. There were 4,867 persons who completed 5 doses of PEP vaccination; 3,051 persons completed PEP within 28 days, the recommended schedule for PEP vaccination.

Of the 6,501 PEP recipients, the disposition of animals that bit humans were not available in 5,955 (92%) at the time of vaccine request; among these, 4,534 (76%) were dogs or cats. There were 399 (8%) dogs or cats reportedly healthy at initial evaluation. Rabies testing was conducted in 149 (2%) animals, including 45 dogs or cats, 17 ferret-badgers, and 42 house shrews. Among them, 12 ferret-badgers and 1 house shrew tested positive for rabies.

As of December 28, 2013, no patients who received PEP had developed symptoms suspected to be associated with rabies.

### Stockpile and distribution of vaccine

**Fig 4:**
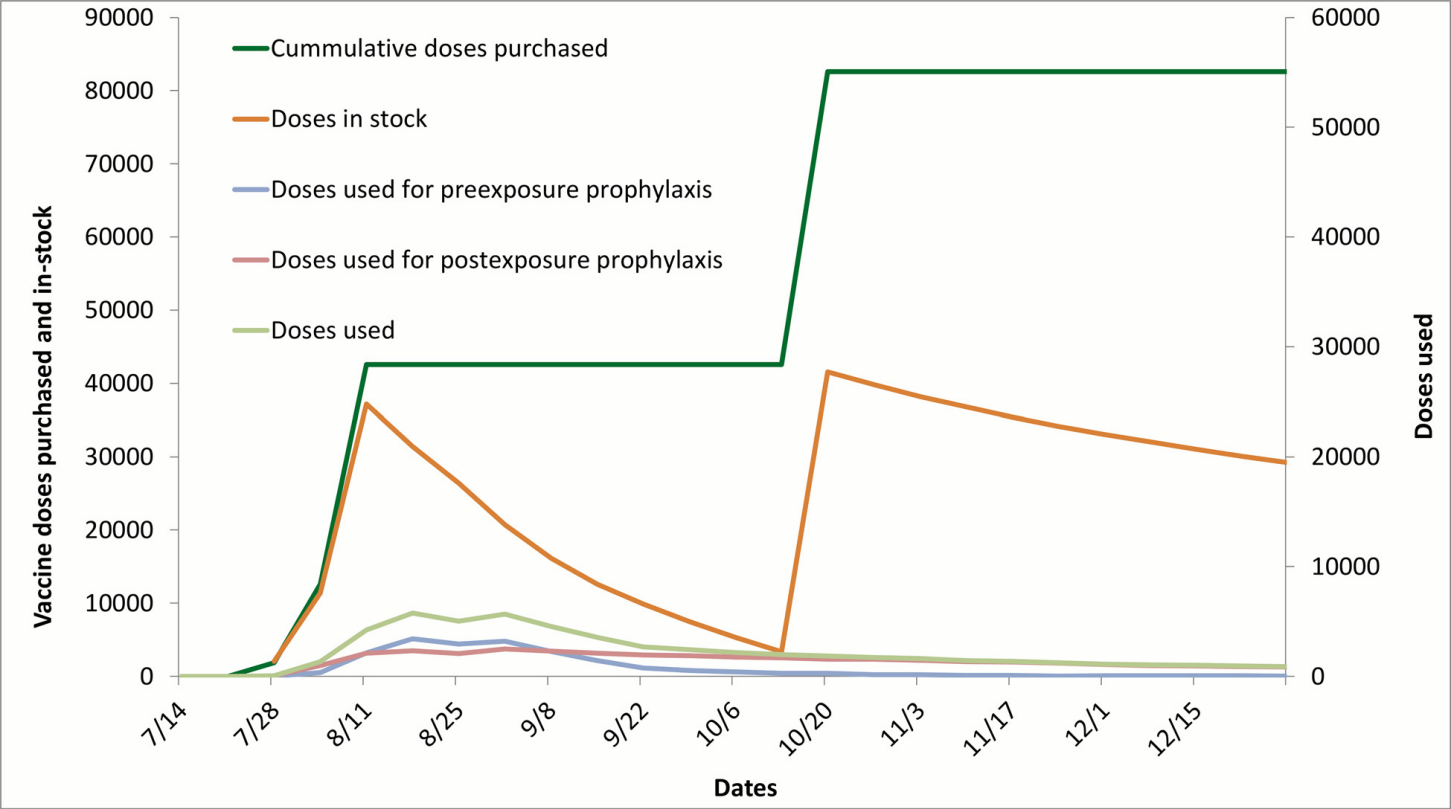
Rabies vaccine and immunoglobulin procurement and use, July 14–December 28, 2013.

With limited rabies vaccine and RIG available at the start of animal rabies reemergence, vaccines were PEP prioritized to persons who sustained bites from wild mammals or stray domestic animals. Vaccine uptake for PEP were low, therefore, vaccines were made available for PrEP. After vaccines were made available for PrEP, vaccine use increased. Vaccine use peaked during week 33, and weekly volume leveled after week 41 (Fig 4). During the study period, there were 6,247 persons who received PrEP, with 18,353 doses used.

### Retrospective identification of ferret-badger exposures

TCDC received 13 reports of ferret-badger exposures that occurred during October 2012 to June 2013. The median age of the 13 patients was 53 years (range 31–71); 10 were male. All the ferret-badger bites were determined to be category III exposures; 10 received RIG and rabies vaccines and 3 received vaccines only.

### AEFI monitoring

As of December 28, 2013, there were 23 reports of AEFI after 48,612 rabies vaccine doses administered (reporting rate 4.7 per 10,000 doses). Of the 23 reports, 4 occurred following PrEP and 19 following PEP; none were classified as anaphylaxis, Guillain-Barré syndrome, or acute disseminated encephalomyelitis. The two SAE reports involved a child, aged 1 year and 11 months, who was hospitalized for acute bronchiolitis 2 days following receipt of the third rabies PEP vaccine dose; and an adult, aged 66 years, who had significant weight loss 6 days following the first rabies PEP vaccine dose and was diagnosed with pulmonary tuberculosis. Among the 21 nonserious reports, the most frequently reported adverse events were rash (n = 8), dizziness (n = 5), and pruritus (n = 4). Seven (30%) patients, including four PEP recipients who reported nonserious adverse events, did not complete the required vaccination series.

### Health education, public health inquiries, and healthcare professional training

As soon as the outbreak of rabies among ferret-badgers was confirmed, TCDC conducted a press conference and posted press release urging the public not to approach unknown animals, especially wild animals. During July 16 to December 28, there were 40,312 calls to the TCDC hotline, of which, 8,692 (22%) were related to rabies (Fig 2). The most common questions concerned PEP and PrEP (n = 5,155, 59%). Others included risk of animal exposure, clinical manifestations of rabies, transmission routes, and where to receive medical care for rabies. Daily inquiries about rabies increased to 115 after the first ferret-badger bit human on July 24, and the peak of calls occurred on July 29. After August 20, daily calls never reached above 100 (mean 21.0).

Healthcare professional training rolled out on July 26. Nationwide, during July 26 to September 1, 2013, there were 21 training sessions conducted, providing healthcare professional updated information on the ferret-badger epidemic, animal bite wound evaluation and management, PEP, and PrEP. These training sessions were hosted by TCDC along with local health departments and medical societies. Over 5,100 persons attended these training sessions, including physicians and nurses in hospitals, central and local health departments, and schools. The decrease in the numbers of call inquiries and PEP not being approved by September may be attributed to intensive public education and training.

## Discussion

As of December 28, 2013, no human rabies cases have been identified in Taiwan through enhanced surveillance despite the continued rabies epizootic among ferret-badgers. However, the newly established mammal bite surveillance demonstrated that bites from stray dogs or cats and wildlife including ferret-badgers, are common and pose increased risk for rabies if PEP is not promptly administered. Swift public health response to rabies reemergence in Taiwan has ensured public awareness of the importance of and access to PEP to prevent occurrence of locally-acquired human rabies cases.

Immediate public health challenges after the first wildlife rabies was confirmed included shortage of human rabies vaccines and RIG as PEP. Prior to confirming Taiwan as a rabies endemic country, few vaccines and RIGs were required countrywide. Therefore, all vaccines and RIGs were procured by TCDC, and then distributed to hospitals as needed. Having the experience of centralized procurement and supply of rabies PEP meant that the emergent procurement process could be pushed forward without having to seek new pharmaceutical firm collaboration, and that TCDC and hospitals already have set standard operating procedure for the distribution of these supplies. Through rapid mobilization of government fund and prompt procurement, TCDC guaranteed the availability of PEP to all who requires vaccines and RIG following animal bites. However, rabies prevention through PEP is costly. A course of vaccination and RIG costs more than USD800, and the US Centers for Disease Control and Prevention estimates that it costs USD10,000 to 100 million for each life saved [37]. Intradermal vaccination protocol has been endorsed by WHO to reduce vaccine use. This protocol has not been endorsed by the national health authorities because intradermal administration of vaccines requires additional training of healthcare personnel, and is not available in all vaccine administration sites. In addition, the vaccine, once opened, must be used up within a few hours, and Taiwan does not have the volume of patients that make intradermal administration cost-saving. Therefore, intradermal PEP protocols were not adopted in Taiwan. The government-funded PEP program has become a financial burden on public funding and sustainability should be taken into account in long-term preparedness plans. As of January 1, 2014, rabies PEP is being offered free of charge through Taiwan’s National Health Insurance scheme, ensuring the sustainable medical care to bite patients, ending the role played by TCDC during the first six month of the animal rabies epidemic.

Because the last case of locally-acquired human and animal rabies cases occurred more than half a century ago, there exists a knowledge gap among the public and medical personnel in Taiwan. Having medical officers trained in multiple disciplines of medicine within TCDC significantly eased the tension during the height of public rabies panic. These medical officers updated the human rabies prevention manual using the latest information, assisted in providing accurate information for public health education, and developed healthcare professionals training materials.

Educating the public on animal-human interaction may reduce animal bites. Furthermore, “catch, vaccinate, sterilize and release” activities and encouraging responsible pet ownership may also decrease stray animals and opportunities for humans to come into contact with unfamiliar animals [38]. Rodent bites consist of 14% of animal bite injuries during our surveillance period. According to the World Health Organization, rodents are usually spillover animals for rabies and do not transmit the disease [15]. Furthermore, rodent bites were often provoked by men trying to rid of rodents from human habitat. Therefore, we must remind citizens to avoid injury from rodent bites when attempting to catch animals.

The peak of PEP requests came shortly after a house shrew was found to be infected by rabies and the revision of the ACIP recommendation for rabies PEP recommendation on the following day. The subsequent panic-driven vaccine-seeking behavior from the public was expected. Because physicians in Taiwan had not been educated about the use of rabies PEP, blanket ACIP recommendation to cover all rodent and shrew bites were unavoidable, even though this is contrary to WHO recommendation. In the ensuing months, physicians treating animal bites must be educated to evaluate bites more thoroughly, and provide vaccines to those who truly require vaccination and RIG. Increased postexposure vaccine-seeking behavior and increased physician delivery of vaccines have been shown to be associated with episodic importation of rabies by Lardon, et al [39]. Media alerts regarding the presence of rabies in the community is shown to be associated with enhanced sense of risk. Following the peak of vaccine-seeking behavior in the wake of the media attention on rabies in Taiwan, PEP use decreased. The reason for the decrease of PEP use in the last two months of our surveillance period is currently unknown. This may have been the result of increased physician awareness of the rabies transmission risk among different animals, decreased medical seeking behavior among patients with animal bites without media stimulation, decreased risky behavior leading to animal bites through public education, or a combination of these causes. High risk personnel receiving PrEP has been advised before rabies was identified in animal population, but uptake of vaccine was low, with approximately 250 doses administered annually. The low uptake is suspected to be because PrEP must be paid by individual patient, while PEP was provided free of charge. With animal rabies reemergence in Taiwan, there is increased perceived risk of illness, thus increased PrEP uptake.

Despite efforts to prevent rabies in humans, rabies control among animals is even more important. Mass canine vaccination and population control is paramount to minimizing the risk transmission of rabies to humans [40]. Following the identification of rabies among ferret-badgers, the Taiwan Council of Agriculture began expanded efforts to provide free rabies vaccination to all owned dogs and cats, especially in rural areas. Stray dogs and cats were also vaccinated and tagged to prevent rabies among animals with frequent human contact. In addition, pets were banned from entering national parks and forested areas where ferret-badgers are known to inhabit, to prevent rabies transmission from ferret-badger to pets. According to a press release from the Bureau of Animal and Plant Health Inspection and Quarantine, Taiwan Council of Agriculture, by March 2014, the 93% of the dogs and cats in areas with rabid ferret-badgers identified were vaccinated, and 79% in areas with ferret-badger activities were vaccinated, and 63% in all other areas were vaccinated [41]. However, on December 29, 2014, a sick civet found in southern Taiwan tested positive for rabies in the same lineage as the rabies virus group from ferret-badger in Taitung [42–44]. Because, to date, only one civet out of the 215 civets tested in 2013 has been found to have rabies [45], it is not yet known if this represent spillover disease from ferret-badger or the virus is now established in the civet population in Taiwan.

There are limitations to this study. Non-medically-attended mammal bites and bites which require no rabies PEP would not have been counted, resulting in underestimation. The true incidence of animal bites is likely to be much higher than currently reported. In addition, PCR testing of CSF is not sensitive for detecting rabies infection [46]. Current strategy in reporting patients with encephalitis of unknown etiologies may also not be sensitive in selecting patients with furious rabies. A more sensitive strategy may be needed to increase the sensitivity of identifying unrecognized human rabies. Because sentinel hospitals submitting specimens for encephalitis of unknown etiology are not geographically representative of Taiwan, human rabies cases may also not have been detected as a result. To ameliorate geographic misrepresentation, on October 28, 2013, TCDC has requested that all hospitals to submit CSF and saliva specimens for rabies testing, if patients have encephalitides of unidentified etiology requiring intensive care or resulting in death. Starting November 2013, rabies virus has been added to the multiplex PCR panel to identify encephalitides of unknown cause.

Because rabies was identified in the ferret-badger population in Taiwan, a previously rabies-free country, the establishment of animal bite surveillance, increased animal testing, emergent RIG and vaccine procurement, and establishment of public education were all important aspects of response. There are still much to be done for the control of animal rabies and prevent human rabies, including studies to characterize interactions between humans and ferret-badgers, establishing improved animal rabies reporting for human disease control, and control of rabies among ferret-badgers. Continued sharing of surveillance, epidemiologic and virologic data between public health and agricultural authorities may facilitate rabies control in both humans and animals. In the long run, continued multifaceted surveillance of animal bites and clinical encephalitides, encompassing both animal and human surveillance, and interministerial collaboration are crucial to achieve the goal of rabies-free status in Taiwan. Continued surveillance is required for countries with and without rabies, to ensure early identification of disease in both humans and animals.
